# Continuous Positive Airway Pressure Versus Nocturnal Oxygen in Obstructive Sleep Apnea: A Propensity Score Matching Study

**DOI:** 10.3390/arm94010008

**Published:** 2026-01-26

**Authors:** Carlos Granados-Burgos, Eduardo Tuta-Quintero, Paula Romero, Laura Gómez-Castro, Alirio Bastidas, Johan Rincón, Sergio Torres, Diego Rodríguez, Kamil Faizal, Juan Moreno, Santiago Monsalve, Estefania Couto, Sofia Yanes, David Torres, Juan Sandoval, Juan Hernández

**Affiliations:** 1 School of Medicine, Universidad de La Sabana, Chía 250001, Colombia; carlosgrbu@unisabana.edu.co (C.G.-B.); johanrihe@unisabana.edu.co (J.R.); charbelfago@unisabana.edu.co (K.F.); juanmoror@unisabana.edu.co (J.M.); santiagomons@unisabana.edu.co (S.M.); estefaniacolu@unisabana.edu.co (E.C.); sofiayaga@unisabana.edu.co (S.Y.); davidtole@unisabana.edu.co (D.T.); juansanroj@unisabana.edu.co (J.S.); juanhepu@unisabana.edu.co (J.H.); 2 Department of Internal Medicine, Universidad de La Sabana, Chía 250001, Colombia; eduardotuqu@unisabana.edu.co (E.T.-Q.); paularoro@unisabana.edu.co (P.R.); lauragomca@unisabana.edu.co (L.G.-C.); sergiotori@unisabana.edu.co (S.T.); diegorodba@unisabana.edu.co (D.R.)

**Keywords:** sleep apnoea, survival, treatment, oxygen therapy, observational study

## Abstract

**Highlights:**

**What are the main findings?**

- Continuous positive airway pressure (CPAP) was associated with significantly higher five-year survival compared with nocturnal oxygen therapy in patients with obstructive sleep apnea.
- CPAP achieved superior control of respiratory events, with a markedly lower residual Apnea–Hypopnea Index than oxygen therapy.

**What are the implications of the main findings?**

- CPAP should remain the preferred first-line therapy for obstructive sleep apnea due to its association with improved long-term survival.
- Nocturnal oxygen therapy may be considered for CPAP-intolerant patients but appears to provide less protection against mortality.

**Abstract:**

Background: Obstructive sleep apnea (OSA) affects quality of life and increases cardiovascular risk. Nocturnal oxygen therapy (NOT) offers a potential alternative for patients intolerant to CPAP. The objective of this study was to compare NOT and continuous positive airway pressure (CPAP) by evaluating five-year survival in patients with obstructive sleep apnea. Methods: A retrospective cohort study was conducted using propensity score matching (PSM) methodology. A PSM analysis was conducted to reduce selection bias due to differences in baseline characteristics between patients using CPAP and those receiving oxygen therapy. Balance between treated and untreated groups was assessed using standardized mean differences. A PSM was estimated using a logistic regression model, matching patients adherent to CPAP therapy to those treated with NOT. Results: A total of 497 patients with a confirmed diagnosis of OSA were included in the analysis. The mean age was 62.1 years (SD13.6), and 54.3% (270/497) were male. Overall, 42.1% (209/497) of the patients were over 65 years old. Of the total, 303 patients received CPAP therapy and 194 received NOT. After PSM, a matched cohort of 370 patients (185 per group) was obtained. The CPAP-treated group showed a significantly lower residual Apnea–Hypopnea Index compared to the oxygen therapy group (3.9, IQR: 1.8–6.5 vs. 15, IQR:7.5–29.1; *p* < 0.001), indicating better physiological control of respiratory events. Treatment with CPAP was associated with a significantly lower risk of mortality compared with NOT across analytical approaches, including weighted logistic regression (OR = 0.11; 95% CI 0.02–0.48; *p* = 0.004) and PSM with bootstrap estimation (ATT = −0.12; 95% CI −0.22 to −0.01; *p* = 0.030). Conclusions: In this cohort, higher five-year survival was observed among patients with OSA treated with CPAP compared with those receiving supplemental oxygen. These findings indicate a favorable association between CPAP use and long-term outcomes, supporting its role as the preferred first-line therapy in patients with OSA.

## 1. Introduction

Obstructive sleep apnea (OSA) has a significant impact on affected individuals due to its detrimental effects on quality of life and its association with increased cardiovascular morbidity and mortality [1,2]. Its overall prevalence ranges from 9% to 38%, being more common in men and older adults [1,2,3]. The treatment of OSA involves a multidisciplinary approach, which includes lifestyle modifications, functional therapy, and the use of medical devices. Continuous positive airway pressure (CPAP) and mandibular advancement devices are established treatment methods [3,4]. Other therapies, such as nocturnal oxygen therapy (NOT), have long been considered an alternative treatment for OSA, particularly in patients who are unable to access or tolerate positive airway pressure [5,6].

Recent studies have highlighted the broader systemic impact of OSA beyond respiratory disturbances. Alterations in circadian clock proteins, such as NPAS2, have been associated with metabolic dysregulation, including insulin resistance, particularly during REM sleep [7]. Additionally, OSA frequently coexists with cardiovascular comorbidities such as arterial hypertension, which is linked to more severe sleep fragmentation, oxygen desaturation, and biochemical alterations, including changes in magnesium and uric acid levels [8]. These findings underscore the complex interplay between OSA, metabolic, and cardiovascular factors, which may ultimately influence long-term outcomes such as survival.

The use of NOT in the management of OSA may have mitigating effects on intermittent hypoxia, reduce the apnea–hypopnea index (AHI) by up to 31%, and improve oxyhemoglobin saturation by approximately 5% [9,10]. Nevertheless, studies directly comparing oxygen therapy to CPAP are limited [4]. Su Latt Phyu et al. [5] conducted a meta-analysis suggesting that NOT could be a potential treatment option for OSA; however, they emphasize the need for additional studies with long-term follow-up and standardized outcome measures [5]. Therefore, the aim of this study is to evaluate the use of NOT in comparison to CPAP and to determine the five-year survival prognosis in patients with OSA.

## 2. Methods

A retrospective cohort study was conducted using propensity score matching (PSM) methodology, including consecutive patients treated at a tertiary care clinic in Colombia between January 2002 and December 2023. This extended inclusion period reflects the real-world availability of long-term follow-up data required to evaluate five-year survival outcomes and ensured an adequate sample size for robust propensity score analyses. All patients who met the predefined inclusion criteria were enrolled in the analysis. The primary outcome was five-year survival, comparing NOT and CPAP use. The reporting of this study adheres to the STROBE guidelines for observational studies [11].

### 2.1. Subjects and Eligibility Criteria

Patients aged ≥18 years with a diagnosis of obstructive sleep apnea (OSA), defined by an apnea–hypopnea index (AHI) >5 events/h and confirmed by polysomnography (PSG), were included. OSA was diagnosed and respiratory events were scored according to the American Academy of Sleep Medicine (AASM) criteria, as defined in the AASM Manual for the Scoring of Sleep and Associated Events [12]. Only patients with CPAP adherence greater than 70% of nights and a mean usage of more than 4 h per night were considered eligible. Patients with central sleep apnea, defined on baseline polysomnography according to AASM criteria as a central apnea index >5 events/h or a predominance of central respiratory events, were excluded. Additionally, patients without available data on oxygen or CPAP use or those lacking follow-up survival data were excluded.

### 2.2. Variables and Data Collection

Data collected included identification and sociodemographic variables such as age, sex, and marital status. Clinical variables included anthropometric measurements obtained during physical examination and relevant medical history extracted from electronic medical records. PSG-related variables analyzed included AHI, apnea type, and the presence of restless legs syndrome. Regarding oxygen therapy, the variables included time since initiation, prescribed daily hours, actual hours used per day, and oxygen flow rate in liters. Treatment indication for speech therapy and variables related to CPAP were also documented. Post-treatment AHI values were obtained from follow-up polysomnography when clinically indicated during routine care. Follow-up PSGs were scored using the same AASM criteria as baseline studies, without modification of hypopnea definitions for either treatment group.

Sleep studies were conducted at the Sleep Laboratory of Clínica Universidad de La Sabana, a tertiary academic referral center in Colombia. All patients underwent standard attended overnight polysomnography performed according to international guidelines, with recordings including airflow, respiratory effort, oxygen saturation, electroencephalography, electrocardiography, and electromyography [12]. Data were acquired and analyzed using commercially available polysomnography systems, and scoring was performed by trained personnel. CPAP therapy was delivered using fixed or auto-adjusting devices from internationally recognized manufacturers widely used worldwide, with pressure titration performed during polysomnography or based on validated clinical protocols [12].

Data were collected from electronic medical records, with follow-up and mortality information obtained from Colombia’s national health information system (ADRES). Clear eligibility criteria were established, and the research team was trained in data collection to reduce the risk of selection and information bias. To minimize data entry errors, data were reviewed by at least two members of the research team.

### 2.3. Sample Size

Sample size was estimated for a cohort study design assuming an event rate of 15% in the exposed group (NOT) and 5% in the unexposed group (CPAP). These assumptions were informed by prior observational literature reporting higher long-term mortality among patients not treated with CPAP, as well as by preliminary institutional data [2,3,5]. A 1:1 allocation ratio, a two-sided alpha level of 5%, and 80% statistical power were assumed. Under these parameters, the minimum required sample size to detect a statistically significant difference between groups was 282 subjects (Machin).

### 2.4. Statistical Analysis

Data were collected using REDCap (Research Electronic Data Capture), version 14.0.18 and analyzed using STATA version 2017 [13]. Qualitative variables were summarized using frequencies and percentages. Quantitative variables with normal distribution were reported as means and standard deviations, and those with non-normal distribution as medians and interquartile ranges. Categorical variables were compared using the chi-square test, and continuous variables were compared using either Student’s t-test or the Mann–Whitney U test, depending on their distribution [14].

A PSM analysis was conducted to reduce selection bias due to differences in baseline characteristics between patients using CPAP and those receiving oxygen therapy. Balance between treated and untreated groups was assessed using standardized mean differences. A propensity score was estimated using a logistic regression model, matching patients adherent to CPAP therapy to those treated with oxygen therapy. The propensity score model included age, sex, body mass index, neck circumference, daytime hypersomnia, smoking status, systemic arterial hypertension, diabetes mellitus, chronic lung disease, and history of cardiovascular disease. Matching was performed using nearest neighbor matching (NNM). Balance before and after matching was evaluated using standardized mean differences and Rubin’s B statistic to ensure comparability between the treated and control groups [11]. After matching, the average treatment effect (ATE) and the average treatment effect on the treated (ATET) were estimated, along with their respective 95% confidence intervals (95% CI) [14]. Kaplan–Meier survival curves were constructed before and after matching. A *p*-value <0.05 was considered statistically significant.

## 3. Results

### 3.1. General Characteristics of the Cohort

**Figure 1:**
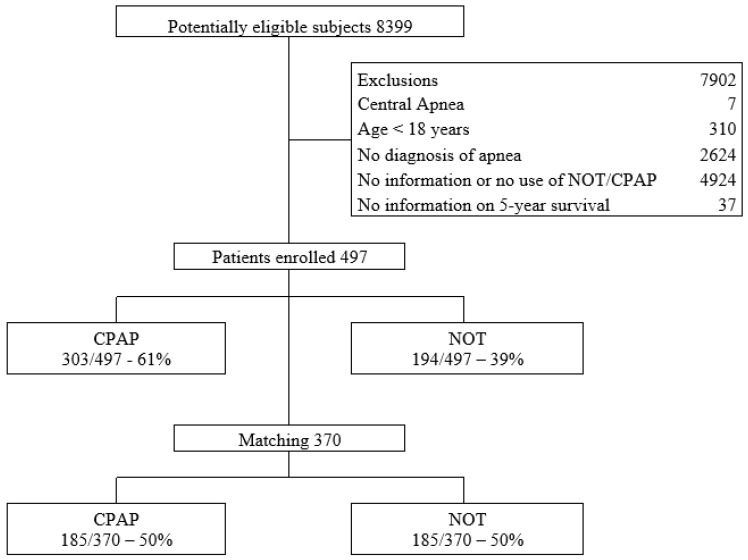
Enrollment flowchart.

A total of 497 patients with a confirmed diagnosis of OSA were included in the analysis. The mean age was 62.1 years (SD 13.6), and 54.3% (270/497) were male. Overall, 42.1% (209/497) of the patients were over 65 years old. Of the total, 303 patients received CPAP therapy and 194 received NOT. After PSM, a matched cohort of 370 patients (185 per group) was obtained Figure 1.

### 3.2. Baseline Comparison Between Groups Before and After Matching

Before matching, significant differences were observed between the groups in key variables such as age (CPAP: 58.8 years, SD: 11.5 vs. NOT: 67.2 years, SD: 15.0; *p* < 0.001) (Table 1). In the original cohort, patients receiving NOT were older and had a significantly higher proportion of individuals aged >65 years compared with the CPAP group (61.3%, 119/194 vs. 29.7%, 90/303; *p* < 0.001). After propensity score matching, both the mean age (62.9 ± 14.9 vs. 60.8 ± 11.2 years; *p* = 0.327) and the proportion of patients aged >65 years (47.5% vs. 35.7%; *p* = 0.105) were substantially balanced between groups, indicating an adequate reduction in age-related baseline differences. Other variables, such as sex, weight, body mass index, and neck circumference, showed minimal differences after matching, with standardized differences below 0.1 in most cases. Regarding symptoms, daytime sleepiness was more prevalent in the CPAP group before matching (48.1%, 143/303 vs. 28.0%, 46/194; *p* < 0.001), but this difference was markedly reduced after PSM (35.7%, 66/185 vs. 37.3%, 69/185; *p* = 0.822).

### 3.3. Comparison of Clinical History

The medical history revealed a high burden of comorbidities, with a prevalence of arterial hypertension of 66.6% (331/497) in the original cohort Table 2. This diagnosis was significantly more frequent in the NOT group before matching (79.9%, 155/194 vs. 58.1%, 176/303; *p* < 0.001), but the difference was reduced in the matched cohort (72.9%, 135/185 vs. 64.9%, 120/185; *p* = 0.255). Other common comorbidities included chronic pulmonary disease (31.2%, 155/497) and major cardiovascular history (25.4%, 126/497). In the original cohort, chronic pulmonary disease was significantly more prevalent in the NOT group compared to the CPAP group (59.8%, 116/194 vs. 12.9%, 39/303; *p* < 0.001); however, this difference was attenuated after matching (27.1%, 50/185 vs. 16.2%, 30/185; *p* = 0.062). Similarly, a history of cardiovascular disease was more common in the NOT group in the unmatched cohort (40.7%, 79/194 vs. 15.5%, 47/303; *p* < 0.001), but the difference also diminished post-matching (23.7%, 44/185 vs. 15.1%, 28/185; *p* = 0.584).

### 3.4. Treatment Adherence and Intermediate Outcomes

The CPAP-treated group showed a significantly lower residual AHI compared to the oxygen therapy group (3.9, IQR: 1.8–6.5 vs. 15, IQR: 7.5–29.1; *p* < 0.001), indicating better physiological control of respiratory events. Adherence-related variables (days of use, proportion of patients using >4 h/day) had incomplete records in the NOT group, limiting direct comparisons in this dimension.

### 3.5. Survival Analysis and Treatment Effect

**Figure 2:**
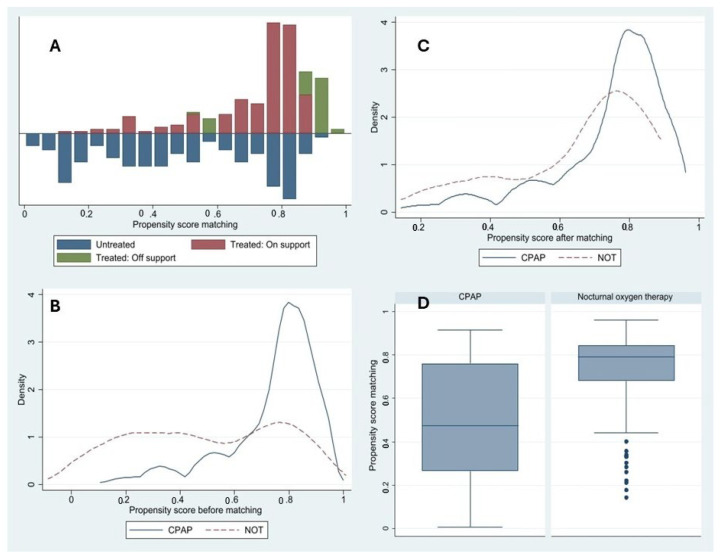
Propensity score matching analysis. Notes: Distribution of the propensity score before (**A**) and after matching between patients treated with continuous positive airway pressure (CPAP) and those who received nocturnal oxygen therapy (NOT). Common support region for both treatment groups before (**B**) and after (**C**) matching. Box-and-whisker plot (**D**) illustrating the distribution of the propensity score in the matched cohort.

**Figure 3:**
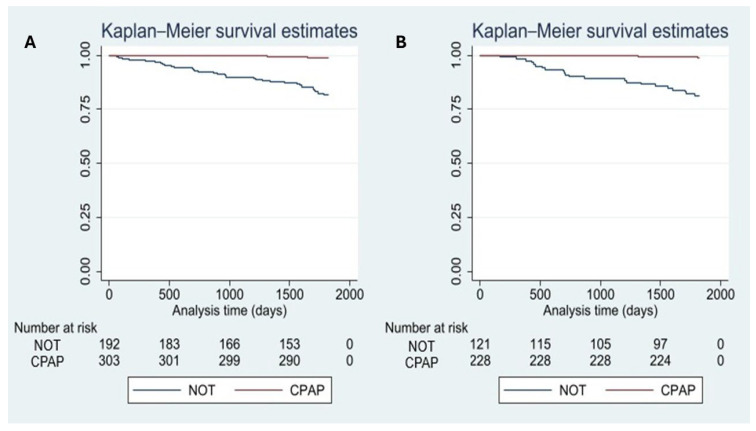
Survival analysis before and after propensity score matching. Notes: (**A**) Survival analysis before matching; (**B**) Survival analysis after matching.

Table 3 presents the results of the PSM using the nearest-neighbor method. As shown in Figure 2A–D, the propensity score distributions demonstrated an adequate common support region between the CPAP and NOT groups. Before matching, a substantial overlap was observed despite differences in the distribution of propensity scores. After matching, the overlap between groups improved, with greater alignment of distributions and reduced dispersion, indicating improved balance and comparability between treatment groups. Covariate balance was assessed using standardized mean differences and Rubin’s diagnostics. Before matching, substantial imbalance was observed (Rubin’s B = 110). After PSM, balance improved markedly, with Rubin’s B decreasing to 37 and Rubin’s R within the acceptable range (R = 1.22), indicating adequate overlap between groups. In the survival analysis (Figure 3A,B), patients receiving NOT exhibited lower survival compared with those treated with CPAP both before and after matching. This difference became more evident after approximately 500 days of follow-up.

## 4. Discussion

In our study, treatment with CPAP was associated with greater five-year survival compared to NOT in patients with OSA. This survival difference was observed among patients adherent to CPAP, defined according to the eligibility criteria as those with usage greater than 70% of nights and a mean use exceeding 4 h per night. It is important to note that patients with treated OSA often present multiple comorbidities, which may contribute to a higher long-term risk of complications. After applying ATE and ATET models, a significant difference favoring CPAP was observed. The distribution of propensity scores before and after matching showed improved overlap between treated and untreated groups, and the Rubin’s index decreased substantially, indicating appropriate covariate balance.

Our findings reinforce and expand the current body of evidence supporting CPAP as the first-line treatment in patients with moderate to severe OSA. In this cohort, CPAP treatment was associated with greater five-year survival compared to nocturnal oxygen therapy, even after adjusting for multiple clinical and sociodemographic variables using propensity score analysis. From a pathophysiological perspective, CPAP corrects the core defect of OSA through the application of continuous positive airway pressure, which stabilizes pharyngeal walls and maintains airway patency [15,16,17,18]. This intervention reduces intermittent hypoxemia, sympathetic activation, intrathoracic pressure swings, and sleep fragmentation—key factors in progressive cardiovascular deterioration—and contributes to the improvement of sleep architecture, cardiovascular function, neuroendocrine regulation, and neurocognitive status [17,18,19,20,21].

NOT, used as an alternative in patients intolerant or contraindicated for CPAP, demonstrated limited benefit in our study [15]. Although adherence was not thoroughly evaluated in the NOT group, our findings emphasize that the survival benefit associated with CPAP at five years was primarily observed in patients with high treatment adherence [15,16,17,18,19]. This underscores the importance of promoting strategies that enhance CPAP adherence, as its efficacy is strongly dependent on therapeutic compliance. Furthermore, before considering alternative therapies such as NOT, it is essential to perform an individualized assessment of the patient’s clinical profile and ability to adhere to CPAP to optimize long-term outcomes.

In our cohort, patients treated with CPAP exhibited a significantly lower residual AHI compared to those who received NOT, reflecting more effective control of the underlying pathophysiological mechanism [16,17,18,19,20]. Unlike CPAP, NOT is limited to improving oxygenation during desaturation episodes but does not prevent the mechanical obstruction of the airway or the associated microarousals [20,21,22]. As a result, patients under NOT maintained an elevated AHI and demonstrated attenuated clinical benefit during long-term follow-up.

Adherence to CPAP treatment is a critical determinant of its clinical benefits, particularly concerning cardiovascular risk reduction and survival improvement. However, objective assessment of adherence can be challenging, as accurate and consistent data on device usage are not always available [23,24,25]. Therefore, a comprehensive evaluation during the initial treatment phase, along with personalized interventions to promote adherence, is a key strategy to optimize long-term outcomes. Prospective studies with standardized follow-up and robust clinical outcome measures are needed to validate these findings and refine the therapeutic algorithm for OSA in real-world clinical settings [15].

Multiple studies have demonstrated that CPAP therapy significantly reduces both systolic and diastolic blood pressure as well as heart rate [25,26,27,28]. Additionally, high adherence to treatment is associated with improvements in mean arterial pressure, reduced daytime sleepiness, lower AHI, and reduced oxygen desaturation index [27,28]. In our study, a history of cardiovascular disease was initially more frequent in the NOT-treated group; however, this difference was no longer statistically significant after matching, suggesting adequate covariate balance between the comparison groups. Patients treated with CPAP experienced improved oxyhemoglobin saturation and reduced daytime sleepiness, as well as a lower incidence of cardiovascular events [1,20,27,28].

### Limitations

Among the limitations of this study is its retrospective observational design, which is inherently susceptible to bias due to unmeasured or incompletely captured confounders. These include socioeconomic factors, undocumented treatment adherence, technical characteristics or adequacy of CPAP devices, and the clinical rationale underlying treatment allocation. Importantly, adherence criteria (>70% of nights and ≥4 h per night) were applied exclusively to the CPAP group to ensure adequate therapeutic exposure, whereas comparable and standardized adherence metrics for nocturnal oxygen therapy were not routinely available. This asymmetry may have introduced selection bias, immortal time bias, and survivor bias favoring CPAP, as inclusion required patients to survive long enough and tolerate therapy sufficiently to demonstrate adequate adherence. In addition, this approach may partially reflect a “healthy adherer” effect, whereby patients adherent to CPAP may also be more likely to adhere to other cardiovascular preventive measures.

Furthermore, nocturnal oxygen therapy is not recommended as a first-line intervention in most clinical guidelines; however, information regarding prior CPAP intolerance, non-adherence, early treatment discontinuation, or other clinical considerations leading to the initiation of oxygen therapy was not systematically recorded. Consequently, treatment allocation may have been influenced by unmeasured clinical or behavioral factors that could not be fully accounted for in the study design. Although a robust propensity score matching strategy was implemented to mitigate baseline imbalances, residual confounding by indication cannot be entirely excluded. In addition, patient-reported outcomes such as perceived sleep quality and longitudinal neurocognitive assessments were not available, limiting the evaluation of functional and patient-centered outcomes.

Nevertheless, a major strength of the study lies in the application of propensity score matching to address substantial baseline imbalances initially present between groups, including an over eight-year difference in mean age and a higher burden of cardiovascular comorbidities in the nocturnal oxygen therapy group. After matching, these differences were markedly reduced, allowing for a more balanced comparison. This methodological approach, together with the assessment of the common support region and Rubin’s diagnostics, enabled the study to approximate the conditions of a randomized clinical trial within an observational framework [14].

## 5. Conclusions

Treatment of obstructive sleep apnea with CPAP is associated with improved five-year survival compared to the use of supplemental oxygen. This finding suggests a clinically meaningful benefit of CPAP as a first-line therapeutic strategy. However, adherence to the device must be considered a key determinant of its effectiveness.

## Figures and Tables

**Table 1:** Population characteristics before and after matching.

	Original Cohort				Matched Cohort				Standardized Absolute Difference	
	Total Population n = 497	NOT n = 194	CPAP n = 303	*p* Value	Total Population n = 370	NOT n = 185	CPAP n = 185	*p* Value	No Matched n = 497	Matched n = 370
Age years, mean (SD)	62.1 (13.59)	67.2 (15)	58.8 (11.51)	<0.001	61.3 (12.24)	62.9 (14.94)	60.8 (11.24)	0.327	0.622	0.157
>65 years n (%)	209 (42.1)	119 (61.3)	90 (29.7)	<0.001	154 (41.6)	88 (47.5)	66 (35.7)	0.105	0.670	0.241
Male sex n (%)	270 (54.3)	97 (50)	173 (57.1)	0.121	217 (58.5)	107 (57.6)	110 (59.5)	0.803	0.143	0.037
Weight kg, mean (SD)	78.8 (15.01)	77.5 (15.27)	79.7 (14.83)	0.110	80.6 (15.36)	81 (16.8)	80.4 (14.92)	0.788	0.147	0.039
Height meters, mean (SD)	1.63 (0.09)	1.61 (0.09)	1.64 (0.1)	0.004	12.2 (149.72)	14.9 (223.34)	11.2 (126.29)	0.474	0.266	0.110
BMI kg/m^2^, mean (SD)	29.7 (5.59)	29.8 (6.28)	29.7 (5.15)	0.764	30.2 (5.84)	30.6 (7.18)	30.1 (5.36)	0.595	0.028	0.085
Symptoms, n (%)										
Daytime hypersomnia	189 (41)	46 (28)	143 (48.1)	<0.001	135 (36.5)	69 (37.3)	66 (35.7)	0.822	0.423	0.034
Insomnia	130 (28.4)	42 (25.9)	88 (29.8)	0.376	120 (32.5)	69 (37.3)	51 (27.7)	0.163	0.087	0.205
Snoring	233 (50.3)	78 (47.3)	155 (52)	0.329	190 (51.5)	103 (55.9)	87 (47)	0.234	0.095	0.179
Tired/fatigued	127 (27.9)	43 (26.4)	84 (28.7)	0.601	107 (29)	56 (30.5)	51 (27.5)	0.653	0.051	0.067
Observed apneas	182 (40.2)	63 (38.4)	119 (41.2)	0.564	171 (46.1)	85 (45.8)	86 (46.4)	0.931	0.056	0.013
Neck diameter centimeters, mean (SD)	41.9 (8.35)	41.5 (4.06)	42.1 (9.39)	0.375	40.8 (4.2)	41.3 (4.75)	40.6 (4.09)	0.296	0.075	0.150

Notes: n: number; NOT: nocturnal oxygen therapy; CPAP: continuous positive airway pressure; SD: standard deviation; BMI: body mass index, expressed in kilograms (kg) per square meter (m^2^).

**Table 2:** Comorbidities before and after matching.

	Original Cohort				Matched Cohort				Standardized Absolute Difference	
	Total Population n = 497	NOT n = 194	CPAP n = 303	*p* Value	Total Population n = 370	NOT n = 185	CPAP n = 185	*p* Value	No Matched n = 497	Matched n = 370
Smoking	207 (41.6)	100 (51.5)	107 (35.3)	<0.001	158 (42.6)	82 (44.1)	76 (41.1)	0.685	0.332	0.060
Consumption > 100 cigarettes	167 (60.9)	83 (65.4)	84 (57.1)	0.165	228 (61.7)	114 (61.8)	114 (61.7)	0.995	0.169	0.001
Systemic arterial hypertension	331 (66.6)	155 (79.9)	176 (58.1)	<0.001	255 (68.9)	135 (72.9)	120 (64.9)	0.255	0.485	0.174
Depression	46 (9.3)	19 (9.8)	27 (8.9)	0.740	43 (11.6)	22 (11.9)	21 (11.4)	0.914	0.030	0.016
Acute myocardial infarction	50 (10.1)	28 (14.4)	22 (7.3)	0.010	27 (7.2)	16 (8.5)	11 (5.9)	0.494	0.232	0.098
Atrial fibrillation	34 (6.9)	22 (11.4)	12 (4)	0.001	22 (5.8)	13 (6.8)	9 (4.9)	0.569	0.282	0.082
Heart failure	69 (13.9)	55 (28.4)	14 (4.6)	<0.001	32 (8.6)	22 (11.9)	10 (5.4)	0.090	0.675	0.231
Peripheral vascular disease	29 (5.8)	15 (7.7)	14 (4.6)	0.149	21 (5.6)	13 (6.8)	8 (4.3)	0.448	0.130	0.107
Cerebrovascular disease	34 (6.8)	22 (11.3)	12 (4)	0.001	19 (5)	13 (6.8)	6 (3.2)	0.233	0.280	0.163
Dementia	34 (6.8)	23 (11.9)	11 (3.6)	<0.001	12 (3.3)	3 (1.7)	9 (4.9)	0.285	0.311	0.179
Chronic lung disease	155 (31.2)	116 (59.8)	39 (12.9)	<0.001	80 (21.7)	50 (27.1)	30 (16.2)	0.062	1.118	0.267
Connective tissue disease	40 (8)	15 (7.7)	25 (8.3)	0.836	31 (8.3)	16 (8.5)	15 (8.1)	0.929	0.019	0.013
Peptic ulcer disease	68 (13.7)	29 (14.9)	39 (12.9)	0.511	43 (11.6)	19 (10.2)	24 (13)	0.568	0.060	0.088
Benign liver disease	43 (8.7)	22 (11.3)	21 (6.9)	0.088	32 (8.6)	19 (10.2)	13 (7)	0.433	0.153	0.112
Diabetes	121 (24.3)	55 (28.4)	66 (21.8)	0.096	80 (21.7)	31 (16.9)	49 (26.5)	0.136	0.152	0.233
Kidney failure	41 (8.2)	24 (12.4)	17 (5.6)	0.008	34 (9.2)	22 (11.9)	12 (6.5)	0.179	0.238	0.187
Cancer	48 (9.7)	21 (10.9)	27 (8.9)	0.469	42 (11.4)	25 (13.6)	17 (9.2)	0.335	0.066	0.138
Traffic accidents	43 (9.1)	24 (13)	19 (6.6)	0.017	39 (10.5)	26 (14.3)	12 (6.7)	0.076	0.218	0.249
Cardiovascular history	126 (25.4)	79 (40.7)	47 (15.5)	<0.001	72 (19.4)	44 (23.7)	28 (15.1)	0.128	0.584	0.218

Notes: n: number; NOT: nocturnal oxygen therapy; CPAP: continuous positive airway pressure.

**Table 3:** Propensity score matching of the effect of continuous positive airway pressure vs. oxygen therapy on 5-year mortality.

Methods	Type of Effect	Coefficient	95% CI	*p* Value
Logistic regression (pweight)	OR	0.106	(0.023–0.484)	0.004
PSM + Bootstrap	ATT	−0.115	(−0.219–−0.011)	0.030
PSM (teffects)	ATE	−0.124	(−0.197–−0.051)	0.001
PSM (teffects)	ATET	−0.092	(−0.183–−0.002)	0.045
PSM + Nearest Neighbor	ATE	−0.124	(−0.197–−0.051)	0.001
PSM + Nearest Neighbor	ATET	−0.092	(−0.183–−0.002)	0.045
NNM (Mahalanobis)	ATE	−0.108	(−0.191–−0.025)	0.011
IPW	ATE	−0.122	(−0.186–−0.059)	<0.001
IPW	ATET	−0.098	(−0.170–−0.025)	0.008

Notes: PSM: Propensity Score Matching; NNM: nearest neighbor matching; IPW: Inverse Probability Weighting; OR: Odds ratio; ATE: average treatment effect; ATET: average treatment effect on the treated.

## Data Availability

The original contributions presented in this study are included in the article. Further inquiries can be directed to the corresponding author.
